# 

**Published:** 1947

**Authors:** 


### The Medical Library of the University of Bristol

WITH WHICH IS INCORPORATED

**The Library of the Bristol Medico-Chirurgical Society.**

---

*The following donations have been received since the publication of the list in the last issue.*

| | |
|---|---|
| Bristol Medico-Chirurgical Society Grant (1) .. .. | 11 volumes. |
| Bristol University, Pharmacology Department (2) .. | 1 volume. |
| Dr. F. M. G. Farrelly (3) .. .. .. .. .. | 1 volume. |
| Dr. G. L. Feneley (4) .. .. .. .. .. | 17 volumes. |
| Institute for the Study of Analgesic and Sedative Drugs (5) .. .. .. .. .. .. | 1 volume. |
| Michell Clarke Fund (6) .. .. .. .. .. | 13 volumes. |
| Munro Smith Fund (7) .. .. .. .. .. | 5 volumes. |
| Royal National Hospital for Rheumatic Diseases, Bath (8) | 1 volume. |
| Yale University (9) .. .. .. .. .. .. | 1 volume. |

Unbound periodicals have also been received from the British Medical Association, Dr. C. D. Evans, Professor T. F. Hewer, Professor R. Milnes Walker and Messrs. John Wright & Sons Ltd.

### THE ONE HUNDRED AND EIGHTY-SIXTH LIST OF BOOKS.

The figures in round brackets refer to the figures after the names of the donors. The books to which no such figures have been attached have either been bought from the Library Fund or received through the Journal.

| | | | |
|---|---|---|---|
| **Blomfield, J.** .. .. .. | *St. George's* 1733–1933 .. .. .. .. .. .. .. | | 1933 |
| **Boyd, W.** .. .. .. | *Surgical Pathology* .. .. .. .. | 6th Ed. (6) | 1947 |
| **Boyd, W.** .. .. .. | *Textbook of Pathology* .. .. .. .. .. | 5th Ed. | 1947 |
| **Buchan, W.** .. .. .. | *Domestic Medicine* .. .. .. .. .. | 13th Ed. (3) | 1792 |
| **Chamberlain, E. N.** .. | *Symptoms and Signs in Clinical Medicine* | 4th Ed. | 1947 |
| **Cochrane, B.** .. .. .. | *An Improvement in the Mode of Administering the Vapour Bath* .. .. .. .. .. .. .. .. .. | | 1809 |
| **Conel, J. L.** .. .. .. | *The Postnatal Development of the Human Cerebral Cortex*, Vol. 2 *Cortex of the One Month Infant* | (6) | 1941 |
| **Cook, Sir E.** .. .. .. | *The Life of Florence Nightingale* .. .. | 2 Vols. | 1913 |
| **Copeman, W. S. C.** .. | *The Treatment of Rheumatism in General Practice* .. .. .. .. .. .. | 4th Ed. (6) | 1946 |
| **Cork, J. M.** .. .. .. | *Radioactivity and Nuclear Physics* .. .. | (7) | 1947 |
| **Crawford, A. M.** .. .. | *Materia Medica for Nurses* .. .. .. | 6th Ed. | 1947 |

| | | | |
|---|---|---|---|
| Denny-Brown, D. | *Diseases of the Basal Ganglia and Subthalamic Nuclei* | (6) | 1946 |
| Dossie, R. | *The Elaboratory Laid Open* | | 1758 |
| Duncum, B. M. | *Development of Inhalation Anaesthesia* | (1) | 1947 |
| Ellis, R. W. B. (Ed.) | *Child Health and Development* | (1) | 1947 |
| Ewart, E. D. | *A Guide to Anatomy* | 6th Ed. (7) | 1947 |
| Falconer, W. | *Observations on Some of the Articles of Diet and Regimen Usually Recommended to Valetudinarians* | | 1778 |
| Fitzwilliams, D. C. L. | *Cancer of the Breast* | | 1947 |
| Galdston, I. | *Progress in Medicine* | (1) | 1940 |
| Goldberg, M. | *English-Spanish Chemical and Medical Dictionary* | | 1947 |
| Grant, J. C. B. | *Method of Anatomy* | 3rd Ed. (1) | 1944 |
| Graves, C. | *The Story of St. Thomas's* 1106–1947 | (1) | 1947 |
| Gross, M. | *Acetanilid. A Critical Bibliographic Review* | (5) | 1946 |
| Grove, A. J., and Newell, G. E. | *Animal Biology* | 2nd Ed. | 1947 |
| Groves, E. W. H. | *Synopsis of Surgery*, 13th Ed., by C. P. G. Wakeley | | 1947 |
| Grüneberg, H. | *Animal Genetics and Medicine* | | 1947 |
| Haggard, H. W. | *The Doctor in History* | (9) | 1934 |
| Hallermann, W. | *Der plötzliche Herztod bei Kranzgefässerkrankungen* | (2) | 1939 |
| Hawk, P. B., and others | *Practical Physiological Chemistry* | 12th Ed. (6) | 1947 |
| Hewer, E. E. | *Textbook of Histology* | 4th Ed. (6) | 1947 |
| Illingworth. C. F. W. | *Short Textbook of Surgery* | 4th Ed. (6) | 1947 |
| Illingworth, C. F. W. | *Textbook of Surgical Treatment* | 3rd Ed. (1) | 1947 |
| Illingworth, C. F. W. and Dick, B. M. | *Textbook of Surgical Pathology*, | 5th Ed. (6) | 1947 |
| Jamieson, E. B. | *Illustrations of Regional Anatomy*, Sections 1, 2. | 7th Ed. (6) | 1947 |
| Jones, E. G. | *An Account of the Eau Medicinale d'Husson in the Gout* | | 1810 |
| Lee, D. H. K. | *The Physiology of Tissues and Organs* | (7) | 1946 |
| Limbourg, J. L. de | *Traité des Eaux Minérales de Spa* | 2nd Ed. | 1756 |
| Macintosh, R. R., and Bannister, F. B. | *Essentials of General Anaesthesia.* | 4th Ed. (1) | 1947 |
| Mackenna, R. W. | *Diseases of the Skin* | 4th Ed. (7) | 1945 |
| McLachlan, A. E. W. | *Handbook of Diagnosis and Treatment of Venereal Diseases* | 3rd Ed. (1) | 1947 |
| Meredith, W. J. (Ed.) | *Radium Dosage—the Manchester System* | (7) | 1947 |
| Metcalfe, R. | *The Rise and Progress of Hydropathy in England and Scotland* | | 1912 |
| Micks, R. H. | *Essentials of Materia Medica, Pharmacology and Therapeutics* | 4th Ed. (6) | 1947 |
| Millin, T. | *Retropubic Urinary Surgery* | (1) | 1947 |
| Muys, J. | *Praxis Chirurgica Rationalis* | | 1684 |
| Pavy, F. W. | *A Treatise on the Function of Digestion, its Disorders and their Treatment* | | 1867 |
| Pye, G. | *A Discourse of the Plague* | | 1721 |
| Robinson, N. | *A New Theory of Physick and Diseases* | | 1725 |
| Robson, J. M. | *Recent Advances in Sex and Reproductive Physiology* | 3rd Ed. | 1947 |
| Schurig, M. | *Lithologia Historico-Medica hoc est Calculi Humani* | | 1744 |
| Stevenson, L. | *Sir Frederick Banting* | (1) | 1947 |
| Stonhouse, J. | *Friendly Advice to a Patient* | 11th Ed. | 1748 |

| | | |
|---|---|---|
| **Strachan, G. I.** .. .. | *Textbook of Obstetrics* .. .. .. .. .. .. .. | 1947 |
| **Tredgold, A. F.** .. .. | *Manual of Psychological Medicine* 2nd Ed. (6) | 1946 |
| **Urbach, E.** .. .. .. | *Skin Diseases, Nutrition and Metabolism* .. (6) | 1946 |
| **Ward, J.** .. .. .. .. .. | *Ad Viri Reverendi C. Middletoni de Medicorum apud Veteres Romanos . . . Dissertationem . . . Reponsio* .. .. .. .. .. .. .. .. .. .. | 1727 |
| **Watkins, A. G.** .. .. | *Paediatrics for Nurses* .. .. .. .. .. .. .. | 1947 |
| **Wofinden, R. C.** .. .. | *Health Services in England* .. .. .. .. .. | 1947 |
| **Young, J.** .. .. .. | *Textbook of Gynaecology* .. .. 7th Ed. (1) | 1947 |

TRANSACTIONS, REPORTS, JOURNALS, ETC.

Reports of the Royal National Hospital for Rheumatic Diseases, Bath. Vol. 1 .. .. .. .. .. .. .. .. .. .. .. .. .. .. (8) 1946

---

### Publications Received

From MESSRS. JOHN WRIGHT & SONS, LTD.:

*Paediatrics for Nurses.* By ARTHUR G. WATKINS, M.D., F.R.C.P. Price 10s. 1947.

*Treatment of Some Chronic and " Incurable " Diseases.* By A. T. TODD, O.B.E., M.B., M.R.C.P. Price 25s. 2nd Edition. 1947.

*British Journal of Surgery,* Volume 35, No. 138. October 1947. Price 12s. 6D. 1947. *Medical Annual* 1947. Price 25s. 1947.

From MESSRS. H. K. LEWIS & CO. LTD.

*The Conduct of Life Assurance Examinations.* By E. M. BROCKBANK, M.B.E., M.D., F.R.C.P. 2nd Edition. Price 12s. 6D. 1947.

### Local Medical Notes

**The Thirty-sixth Long Fox Lecture** was given on 18th November, 1947 by Professor Hadfield, M.D., F.R.C.P., on " **Sub-acute Bacterial Endocarditis.**" The members of the appointing board did not escort the lecturer. Professor Hewer was in the chair. 53 persons attended the lecture.

---

As we go to press, we learn with deep regret of the death of Mr. T. Carwardine. An Obituary notice will appear in the next issue.

EXAMINATION RESULTS.

**University of Bristol.**—Members of the University have passed the following examinations :—

M.D.—D. N. Walder.

M.B., Ch.B.—*Second Examination* (*Section II*) *:* S. H. Brown, Megan D. M. Johnson.

M.B., Ch.B.—*Final Examination :* Margaret J. Andrews, D. H. Bowden, D. M. Cunningham, Eudora M. R. Davies, N. M. Gibbs, Pamela H. L. Hamilton, S. F. Iles, Jean E. Morrell, Josephine C. Mulcahy, J. P. Norris, P. W. Seargeant. *In Group I* (*completing the Examination*) *:* Catherine M. Hoyle. *In Group II* (*completing the Examination*) *:* C. E. Halliday, Kathleen C. Iles.

M.B., Ch.B.—*Final Examination* (*Section I*) *:* C. C. Downie, Margaret Ford, Anne M. French, G. A. Hanks, Joan A. Higginbottom, Elizabeth Preston-Thomas, P. J. Speller, G. D. Teague, B. F. Vaughan, Pamela L. C. Watson, Maro A. Wells, R. H. Wood.

B.D.S.—*Second Examination :* M. Bernstein.

B.D.S.—*Third Examination. In Dental Mechanics* (*completing the Examination*) *:* D. C. Berry.

B.D.S.—*Final Examination. In Section II* (*Dental Surgery*) *completing the Examination :* R. J. Brownlee, L. K. Walden.

L.D.S.—*Third Examination :* G. D. Pamely. *In Dental Mechanics only :* G. D. Everard, H. T. Jones.

L.D.S.—*Final Examination. In Section II* (*Dental Surgery*), *completing the Examination :* J. C. Fishpool. *In Section I only :* M. J. Bartlett, D. Clifford, L. B. Howell, H. T. Ivory, Margaret J. Webb.

D.P.H.—*Part I :* Roma N. Chamberlain, J. B. M. Davies, D. M. M. Jones, Brenda G. King, E. W. Moore, W. Nicol.

D.P.M.—*Part I :* V. G. Crotty, R. M. Ellison, J. T. Robinson. *In Anatomy and Physiology of the Nervous System (completing the Examination) :* I. M. Davies. *In Psychology (completing the Examination) :* A. Esterman, I. A. Macdonald, R. Maggs, H. J. L. O'Sullivan.

D.P.M.—*Part II :* L. B. Thomas.

### Bristol Medico-Chirurgical Journal.

Published Quarterly (royal 8vo. reduced) under the auspices of the Bristol Medico-Chirurgical Society.

---

*An excellent means of advertising.*

---

The only Medical Journal published in the West of England, and has an extensive circulation not only amongst the Medical men of Bristol, but also amongst those of the adjoining Counties and South Wales, and in exchange goes to all parts of the world.

Terms for Advertisements:
Whole Page, £2 2s.; Half Page, £1 5s.; Quarter Page, 15s. per issue.

PRICES FOR SPECIAL POSITIONS ON APPLICATION.

J. W. ARROWSMITH LTD.,
Advertising Agents,
Quay St. & Small St., BRISTOL.

